# Parliament and the Profession

**Published:** 1916-12-09

**Authors:** 


### PARLIAMENT AND THE PROFESSION.

### Venereal Diseases amongst Home Forces.

MR. FORSTER informed Mr. Houston that the admissions to hospitals at home of men in the Army suffering from enthetic (venereal) diseases represent a ratio of 48 cases per 1,000 men per annum, which is slightly less than the ratio in peace time; no figures are available for troops abroad. No men are discharged from the Army for these diseases in the infectious stage.

### Hospital Conditions in India.

MR. CHAMBERLAIN, when answering several questions as to the alleged unsatisfactory condition of some Indian hospitals, complained of the frequent anonymity of the allegations. The most recent complaint was in respect of Colaba Hospital, and referred to bed linen, pyjamas, and other comforts, and to a plague of bugs. The General commanding at Bombay had associated himself with an inquiry, during which it was elicited that no complaints had been entered in the book kept for the purpose in the officers' ward. Bugs had been discovered, and every means were being taken to eradicate them; there was an ample supply at all times of clean linen and other comforts.

### Medical Recruiting Fees.

MR. COWAN, in a question to the Secretary of State for War, sought information as to the varying rates paid to doctors for examining recruits. Mr. Forster said that £2 is considered a fair maximum for a full day's work, and authority to exceed this sum had only been given in exceptional cases where a rate per head, without limitation as to total, had been promised, but it is not considered that a similar arrangement in other cases can be justified.

### National Insurance Certificates.

MR. O'GRADY having asked whether, having regard to the scarcity of qualified doctors, senior students in their final year could not be allowed to sign medical insurance certificates on behalf of their principals, Mr. Roberts pointed out that this matter was governed by the principle that the responsibility for giving certificates could not be diverted from the responsibility for diagnosis which a medical practitioner is professionally debarred from delegating to an unqualified man.

### Venereal Diseases.

THE President of the Local Government Board informed Mr. Pratt that the regulations issued by his Department in respect of the diagnosis and treatment of venereal diseases empower local authorities to make provision for the giving of instructional lectures and for the publication of information on questions relating to these diseases. The circular which accompanies the regulations draws attention to the contents of the report of the Royal Commission.

